# Self-Assembly Properties of Cationic Gemini Surfactants with Biodegradable Groups in the Spacer

**DOI:** 10.3390/molecules24081481

**Published:** 2019-04-15

**Authors:** Martin Pisárčik, Mája Polakovičová, Mário Markuliak, Miloš Lukáč, Ferdinand Devínsky

**Affiliations:** 1 Department of Chemical Theory of Drugs, Faculty of Pharmacy, Comenius University, Kalinčiakova 8, 83232 Bratislava, Slovakia; markuliak1@uniba.sk (M.M.); lukac@fpharm.uniba.sk (M.L.); 2 Department of Pharmaceutical Chemistry, Faculty of Pharmacy, Comenius University, Odbojárov 10, 83232 Bratislava, Slovakia; maja.polakovicova@uniba.sk; 3 Faculty of Pharmacy, Comenius University, Odbojárov 10, 83232 Bratislava, Slovakia; devinsky@fpharm.uniba.sk

**Keywords:** gemini surfactant, micelle aggregation number, biodegradable spacer, time-resolved fluorescence quenching, micelle size, zeta potential

## Abstract

Self-assembly properties of cationic gemini surfactants with biodegradable amide or ester groups in the spacer were investigated utilising time-resolved fluorescence quenching, dynamic light scattering and zeta potential measurements. A correlation between aggregation parameters such as micelle aggregation number, micelle size and zeta potential with the structure of gemini molecules was made. For gemini molecules with medium spacer lengths, micelle aggregation number does not change much with the surfactant concentration. When the spacer is extended, a stronger aggregation tendency is observed for gemini surfactant molecules with two ester groups in the spacer and the aggregation number increases. The assumption of stronger aggregation of ester-based gemini molecules at larger spacer number values is also documented by measurements of the size and zeta potential of ester-based micelles. The explanation of the difference in aggregation ability of amide-based and ester-based gemini molecules is related to the structural features of gemini molecules, notably to the larger flexibility and denser arrangement of ester-based gemini molecules in a micelle. To support this assumption, optimised 3D models of the studied gemini molecules were constructed. Correspondingly, the calculations show smaller size and interfacial area for ester-based gemini conformers.

## 1. Introduction

Gemini or dimeric surfactants, composed of two hydrophilic heads, two hydrophobic chains and a spacer interconnecting the hydrophobic moieties, have attracted research interest over several decades. They show potent surface activity [1], low critical micelle concentration (*cmc*) [2,3] and provide variety of applications in different industrial branches (e.g. catalysts, viscosity modifiers, participants in polymerisation processes, etc.) [4,5,6,7]. A special class of surfactants, so-called “soft surfactants” [8], contains chemical groups in their molecular structure which makes them biodegradable. These groups provide surfactant molecules with biodegradable properties by decomposing long hydrocarbon molecule parts into shorter ones. Among various functional groups, gemini surfactants with ester or amide groups are of special importance. Usually, the weaker ester or amide bond is hydrolysed, and the surfactant molecule is decomposed into smaller products in terms of molecular size and weight. A substantial part of research interest is devoted to gemini surfactants with ester groups. They can be located either in the hydrophobic tails of gemini molecules [9,10,11,12,13] or in their spacers [14,15,16,17,18,19,20,21,22]. It was found that the presence of ester bonds in the gemini surfactant molecular structure affects the physico-chemical and aggregation properties of these surfactants, e.g. it increases the hydrophilicity and aqueous solubility of gemini species [9,22], changes the *cmc* and increases the micellar solubilisation capacity [14,15]. More pronounced morphological changes of aggregates composed of gemini surfactants with diester groups in the spacer were found for surfactant molecules with longer tail lengths (16 carbon atoms) [17,18]. The changed spacer rigidity represented by the presence or absence of a double bond between two ester groups affects the surface and aggregation properties of these gemini molecules [19]. Biodegradability studies on ester-based gemini surfactants [16,22] revealed that the location of the ester group in the gemini spacer is more favourable for biodegradation as compared with ester groups located in the two hydrophobic tails of a gemini molecule [16]. Studies with bisammonium gemini molecules containing ester groups as a part of hydrophobic tails showed that the shape of their aggregates varies from oblates to tablet-shaped micelles depending on their spacer length [12]. They were also found to efficiently solubilise organic dyes. However, the presence of ester bonds in the alkyl chains reduces the solubilisation power [10]. The synthesis of cationic ester-based gemini surfactants is not just confined to gemini molecules with ammonium hydrophilic parts. Bisimidazolium gemini surfactants with variable length of the alkyl chains containing two ester groups and a constant spacer length showed very low *cmc* and a good efficiency in reducing the surface tension of water [13]. Ester-based gemini surfactants also show potent biological activity such as antimicrobial activity and cytotoxicity [20,21]. Research interest is also devoted to cleavable gemini surfactants bearing amide groups which are located either in the gemini spacer [23,24] or are part of hydrocarbon tails [25,26,27]. Synthesis and physicochemical investigations of novel cationic gemini surfactants with diamide spacers are reported [23,24]. It was found that the aggregation properties of gemini molecules with amide groups strongly depend on the position and number of the amide groups [26]. Several aggregation parameters such as micelle diameter and aggregation number, strongly depend on the molecular structure of gemini surfactants. Micelle aggregation number increases with the number of amide groups in gemini molecule [26] or, in case of the location of amide groups in surfactant tails, with surfactant concentration [25]. The determination of micelle aggregation number for aggregates composed of ester-based gemini surfactants revealed that the value of the aggregation number is primarily controlled by the length and the nature of the spacer and, to a lesser extent, by the length of the alkyl chains [10]. In our previous study, we analysed the aggregation behaviour cationic bisammonium gemini surfactant molecules with dodecyl tails and a polymethylene spacer utilising the time-resolved fluorescence quenching experimental technique [28]. The goal of the present paper is to investigate aggregation properties of cationic gemini surfactants with biodegradable amide or ester groups in a spacer of variable length utilising the determination of micelle aggregation number, micelle size and zeta potential. Along with the analysis of the above-mentioned aggregation parameters of gemini micelles, a simple correlation of the gemini spacer molecular structure utilising the construction of optimised 3D models of gemini molecules with micelle aggregation number values is provided.

## 2. Results and Discussion

### 2.1. Diamide and Diester Gemini Surfactants

**Scheme 1:**
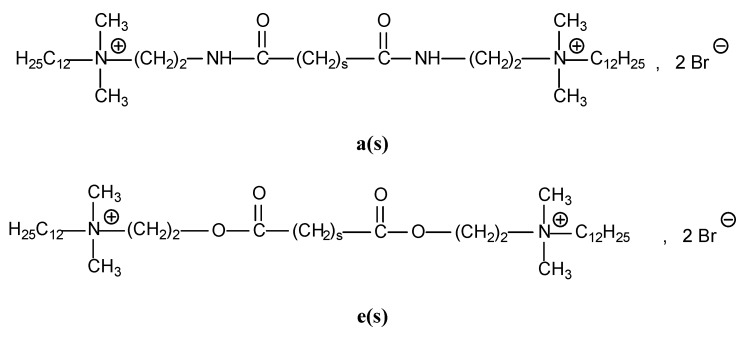
Chemical structures of **a(s)** and **e(s)** gemini surfactants. Surfactant spacer number **s** varies between 1 and 8 CH_2_ groups.

Two series of cationic bisammonium gemini surfactants with two dodecyl chains and a diamide spacer (hereinafter referred to as **a(s)** where **s** is the number of carbon atoms in the spacer central part) and with two dodecyl chains and a diester spacer (hereinafter referred to as **e(s)** where **s** is the number of carbon atoms in the spacer central part), were synthesized. The diamide geminis **a(s)** were prepared from bis-(2-dimethylaminoethyl)amide of the corresponding α,ω–alkanedicarboxylic acid which was subsequently quaternised with 1-bromododecane. The products were purified by repeated crystallisation from anhydrous methanol or methanol-ether mixtures. The diester geminis **e(s)** were synthesised by the reaction of alkane-α,ω–diylbis(bromoethylacetate) with dimethyl-dodecylamine. The products were purified by crystallisation from anhydrous acetone-methanol mixture. Gemini surfactants were synthesised with the variable number of carbon atoms **s** in the spacer central part. The formulas are shown in Scheme 1 for both the **a(s)** and **e(s)** series. The critical micelle concentrations (*cmc*) for both the **a(s)** and **e(s)** series determined from surface tension measurements are shown in Table 1.

### 2.2. Micelle Aggregation Number of Diamide Gemini Surfactants

**Figure 1:**
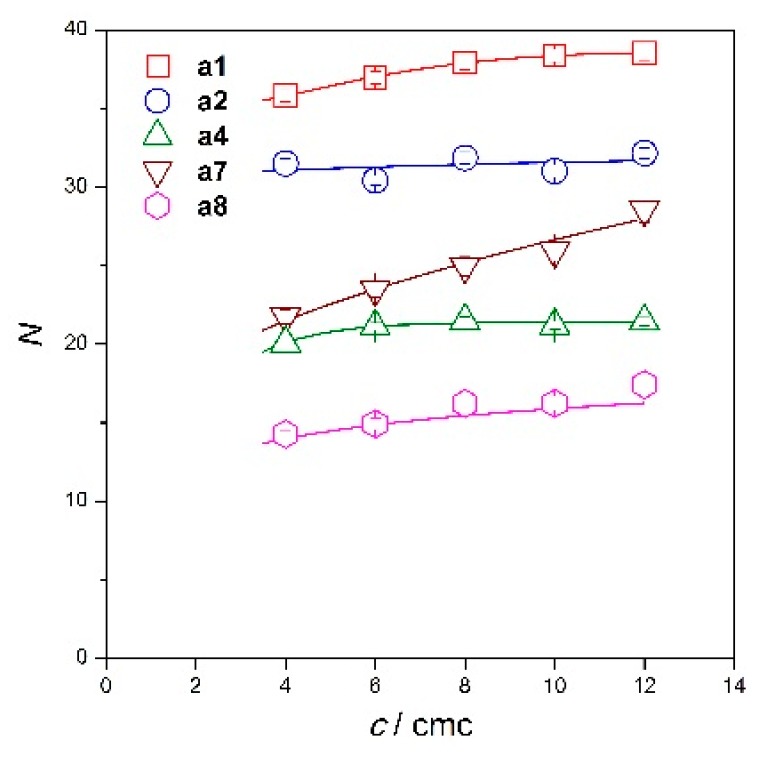
Micelle aggregation number *N* of plotted as a function of concentration *c/cmc* for spacer values **s** of diamide gemini surfactants **a(s)**. The solid curves are a guide to the eye.

In Figure 1, the aggregation number of micelles of diamide gemini surfactants **a(s)**, as determined from fluorescence decays of time-resolved fluorescence quenching, is plotted for different spacer values **s** as a function of gemini surfactant concentration. 

The fitting constant *R* from the fluorescence decay measurements and the aggregation number *N* of **a(s)** micelles calculated from Equation (2) in Section 3.2 are listed in Table 2 for all concentration values *c*/*cmc* and spacer values **s**.

**Figure 2:**
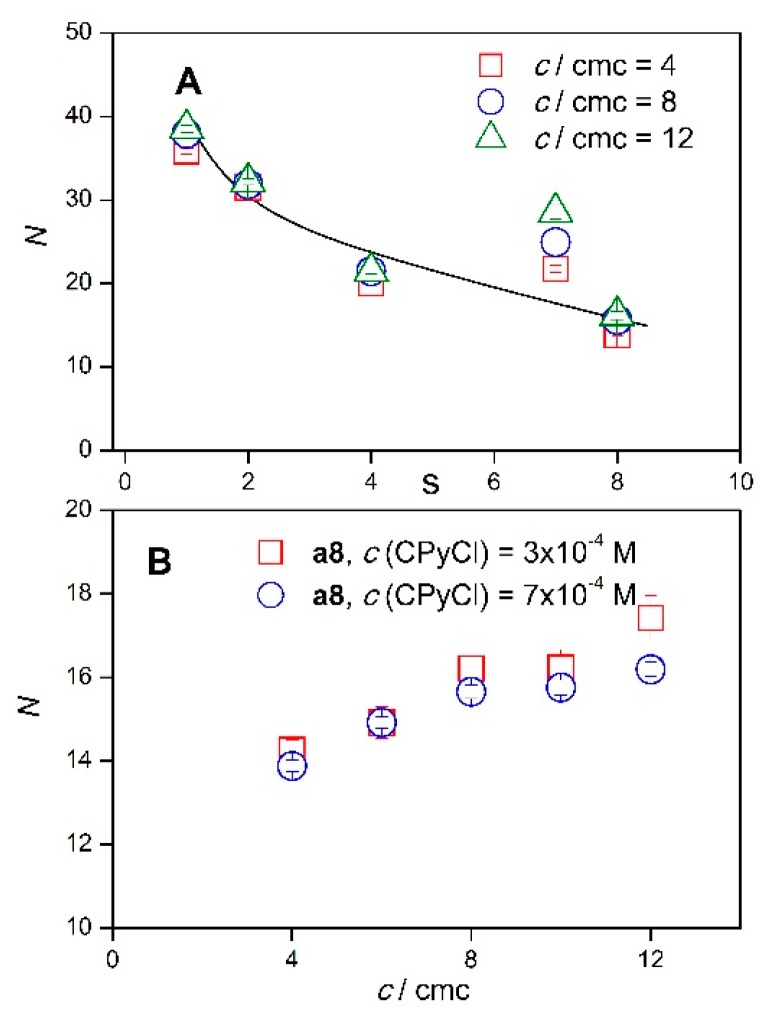
**A**: Micelle aggregation number *N* of **a(s)** surfactants plotted as a function of spacer number **s** for three concentrations *c/cmc* = 4, 8, 12. **B**: Micelle aggregation number *N* of **a8** surfactant plotted as a function of surfactant concentration *c/cmc* for two quencher concentrations. The solid curve is a guide to the eye.

Micelle aggregation number values of diamide gemini surfactants with variable spacer length **a1**–**a8** were found to be in the range 15–38, depending on surfactant concentration. As seen from Figure 1, micelle aggregation number values for diamide surfactants **a1** to **a8** almost do not depend on surfactant concentration *c*/*cmc*. This implies the formation of stable spherical micelles with a constant aggregation number for diamide geminis **a(s)**, **s** = 1-8 in the concentration range 4 × *cmc* to 12 × *cmc*. In Figure 2A, the plot of *N* vs. spacer length **s** is shown for three different surfactant concentrations *c*/*cmc* = 4, 8, 12. 

It results from the plot 2A that the aggregation number increases with the decreasing spacer length from 8 to 1 CH_2_ group in the **a(s)** spacer central polymethylene part separating the two amide groups (Figure 2A). This underlines the increased aggregation ability of diamide gemini surfactants as the spacer length decreases. The decrease in methylene groups number from 8 to 1 in the spacer central part results in doubling the aggregation number value (from 15 to 38). A similar effect was observed for bisammonium gemini surfactants with dodecyl chains and a polymethylene spacer **12-s-12** within our previous study of determination of micelle aggregation number for these gemini surfactants [28]. The concentration increase in **a1** and **a2** micelle aggregation number (Figure 1 and Figure 2A) is to relate to the aggregation properties of these surfactants which are controlled by the gemini spacer length. Previous studies indicate a nonlinear dependence of aggregation parameters (interfacial area per surfactant molecule, micelle aggregation number, particle diameter) on the spacer length. Increased aggregation is reported for very short spacers composed of two, three CH_2_ groups (formation of nonspherical, rodlike micelles) and for long flexible spacers with 10 and more CH_2_ groups (large micelles and vesicles for spacers with the length exceeding 16 CH_2_ groups). The medium spacer length (four or five CH_2_ groups) represents the area of rigid spacers which are unable to be bent into micelle hydrophobic core. This results in the formation of small spherical micelles with small hydrodynamic diameter, small aggregation numbers independent of surfactant concentration and large interfacial area per surfactant molecule [29,30]. A similar observation was also made in our previous study of aggregation of urea-based gemini surfactants [31] where nonlinear dependences of aggregation parameters were found as a function of the spacer length with worse aggregation detected for medium spacer lengths. Moreover, strong aggregation for short spacer gemini molecules with urea groups in the alkyl chains also resulted from the increased hydrogen bonding between urea groups [31]. This could also be the case of gemini molecules with short spacer (**a1, a2**) where the amide groups in the spacer are separated by just one or two methylene groups and intramolecular hydrogen bonding can take place which results in the increased aggregation number values (Figure 1 and Figure 2A). Figure 2B shows the influence of quencher concentration on the determination of micelle aggregation number of diamide gemini surfactants. For **a8** micelles, the aggregation number was determined at two different quencher (CPyCl) concentrations (3 × 10^−4^, 7 × 10^−4^ M). In both cases, the calculated *N* values are practically identical which indicates that aggregation number is independent of quencher concentration in the investigated quencher concentration range. 

### 2.3. Micelle Aggregation Number of Diester Gemini Surfactants 

**Figure 3:**
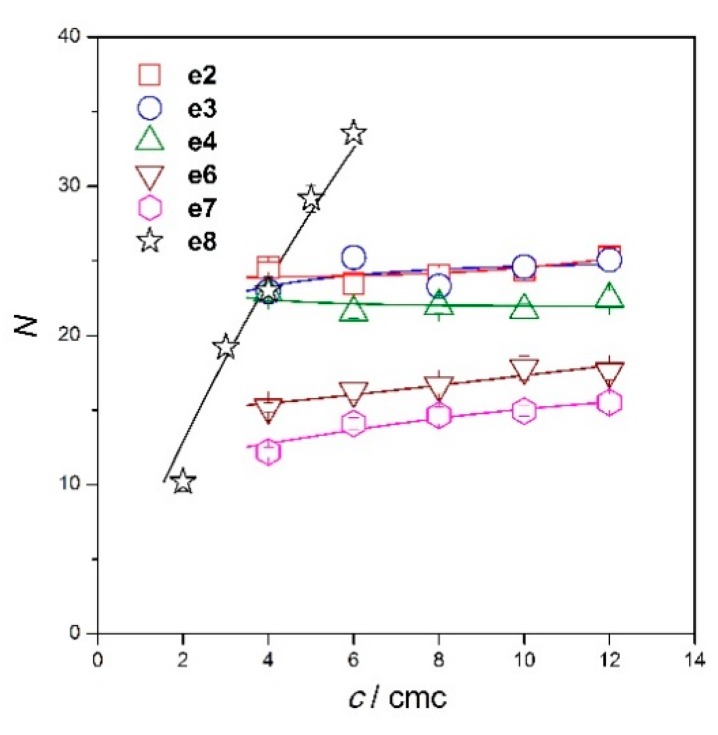
Micelle aggregation number *N* of plotted as a function of concentration *c/cmc* for spacer values **s** of diester gemini surfactants **e(s)**. The solid curves are a guide to the eye.

Concentration dependence of micelle aggregation number of diester gemini surfactants **e(s)** is shown in Figure 3 and the aggregation number values are listed in Table 3 for all concentration values *c*/*cmc* and spacer values **s**.

Micelle aggregation number values of **e2** to **e7** diester surfactants were found to be in the range 12–25 (Figure 3, Table 3). As observed with the **a(s)** series, the dependence of micelle aggregation number of **e2** to **e7** gemini surfactants on concentration *c*/*cmc* is again moderate, which implies the presence of stable spherical micelles for this range of spacer lengths. A different aggregation behaviour was observed for gemini molecules with the longest spacer **e8**, where a steeper increase of micelle aggregation number with surfactant concentration occurs (Figure 3). It results from the plot that the maximum micelle aggregation number is equal to 33 (Table 3), was reached for **e8** at just a concentration 6 × *cmc* which is half of the maximum concentration 12 × *cmc* of other diester gemini surfactants **e(s)**.

**Figure 4:**
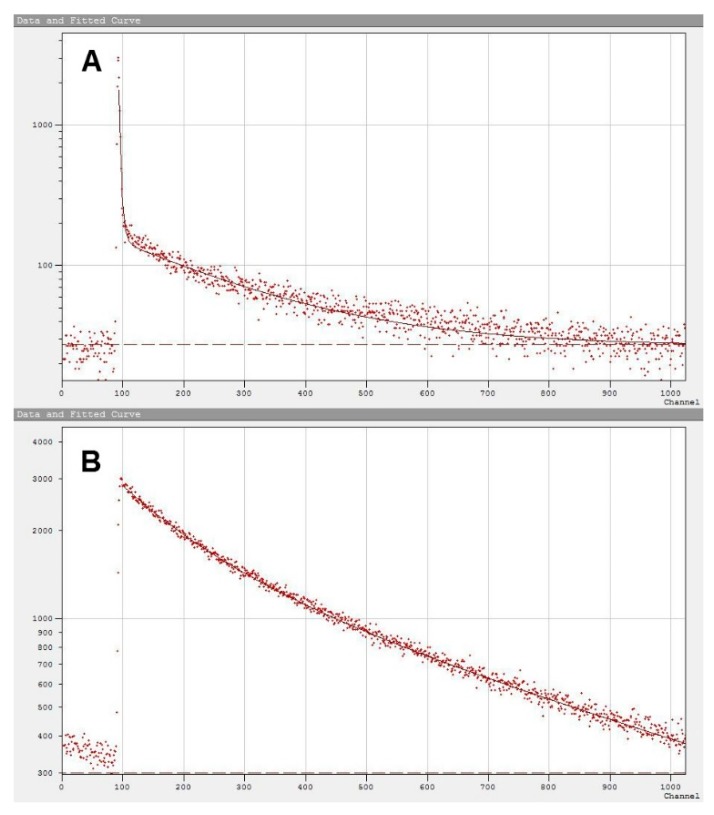
Fluorescence decays for **A**: **e8**, **B**: **e4** surfactants. For both surfactants, *c*/*cmc* = 6. Pyrene concentration is 4 × 10^−6^ M (**e8**), 1 × 10^−5^ M (**e4**), quencher concentration is 1 × 10^−4^ M (**e8**), 4 × 10^−4^ M (**e4**). The solid curves are results of the non-linear fit according to the Equation (1).

**e8** solutions at the concentrations larger than 6 × *cmc* were also prepared, but they were unstable and phase separation occurred immediately after the preparation. This indicates a stronger aggregation tendency of **e8** which is to relate to the **e8** molecular structure as explained later in Section 2.5. It is also interesting to note that this steep concentration increase in *N* was not observed for **a8** diamide gemini surfactant of identical spacer length (Figure 1). This observation is to relate to different ester and amide bond properties, as explained utilising 3D models of **a8** end **e8** molecules in the Section 2.5. The increased aggregation behaviour of **e8** is also demonstrated by the fluorescence decay shape different from that observed for a gemini surfactant with the weak concentration dependence of aggregation number, e.g., **e4** (Figure 4). 

Figure 4A shows the initial fall-off of the decay curve which results in a large value of the constant *R* and hence, the aggregation number *N* of **a8** micelles. In contrast, the decay curve for **e4** (Figure 4B) does not show any initial steep decrease. Consequently, the fitting constant *R* and aggregation number *N* are smaller than those of **a8**. This observation would assume the formation of vesicles composed of **e8** gemini molecules. Surfactant vesicles are usually composed of gemini molecules with a very long spacer (20 atoms and more) or based on the spontaneous interaction of cationic and anionic surfactants [30,32]. However, we believe that the aggregation number values for **e8** (Table 3) are too small to form a closed surfactant double layer (vesicle). 

### 2.4. Micelle Size, Zeta Potential Determination

**Figure 5:**
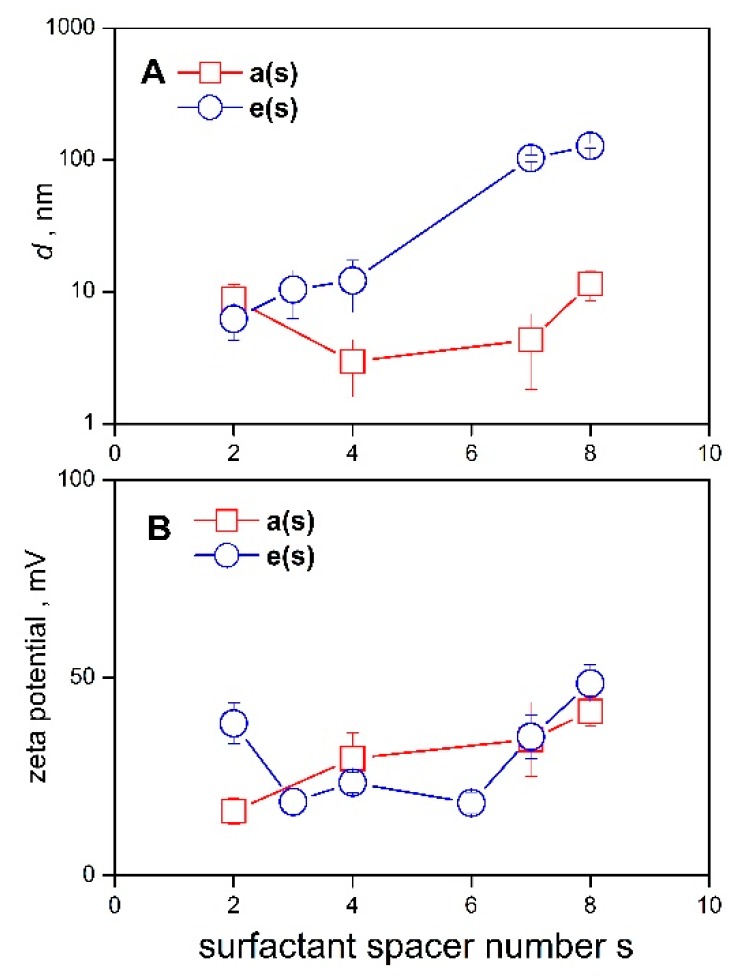
**A**: Micelle hydrodynamic diameter *d* of **a(s)** and **e(s)** gemini surfactants plotted as a function of surfactant spacer number **s**, at the surfactant concentration 6 × *cmc*. **B**: Zeta potential of **a(s)** and **e(s)** gemini micelles plotted as a function of surfactant spacer number **s**. Zeta potential of **a(s)** and **e(s)** gemini micelles was determined at the surfactant concentration 6 × *cmc* and 15 × *cmc*, respectively.

Mean micelle hydrodynamic diameter of both series of gemini surfactants **a(s)** and **e(s)** was determined utilising dynamic light scattering method and is plotted in Figure 5A as a function of surfactant spacer number **s**. The surfactant concentration for all samples was 6 × *cmc*. 

Upon the mutual comparison of micelle diameters of **a(s)** and **e(s)** surfactants, a stronger aggregation tendency of diester molecules **e(s)** is observed, as documented by the larger particle size values and a steeper concentration increase of **e(s)** particle size (Figure 5A). The increase in the mean hydrodynamic particle diameter for long spacers of **e7** and **e8** diester surfactants may indicate the formation of intermicellar aggregates because their aggregation number is not large enough to form vesicles, as it is discussed in the previous section. In contrast, micelle size of diamide micelles **a(s)** lies below or close to 10 nm which indicates the formation of spherical micelles in the whole range of **s** values of **a(s)** diamide gemini surfactants. 

**Figure 6:**
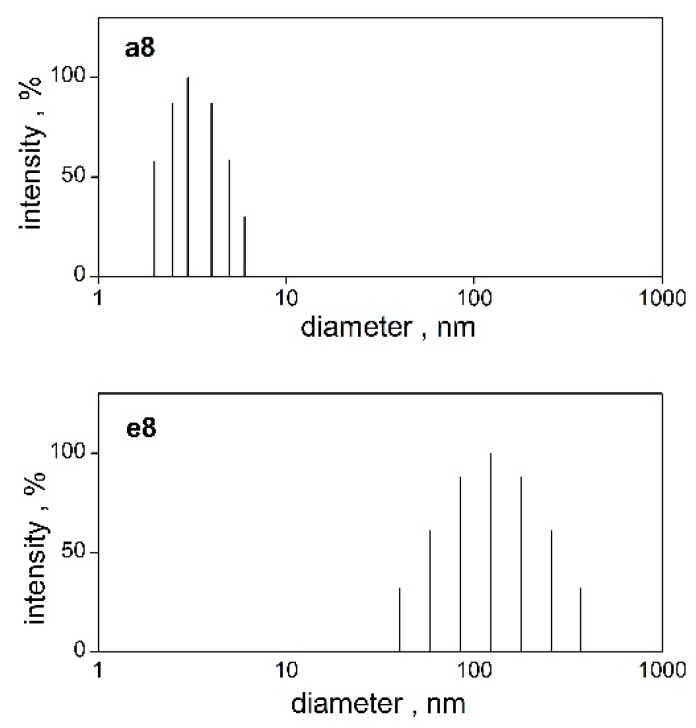
Particle size spectra obtained from the time correlation functions using the CONTIN algorithm for **a8** and **e8** gemini surfactants.

Particle size spectra of **a8** and **e8** micelles (Figure 6) obtained using the CONTIN algorithm applied on the time correlation functions show the peaks at the particle diameter values 3 and 122 nm for **a8** and **e8** surfactants, respectively (Figure 6). 

It is interesting to note that the peak responsible for the formation of small spherical micelles of the size of several nanometers is not observed for **e7** and **e8** gemini molecules in the particle size spectra. The large value of **e8** aggregate size around 100 nm may indicate the presence of some form of intermicellar aggregates. The formation of vesicles may be excluded because of the low values of aggregation number, as discussed in Section 2.3.

The zeta potential of micelles of **a(s)** and **e(s)** gemini surfactants was determined at the concentration 6 × *cmc* and 12 × *cmc*, respectively. The conductivity required for the zeta potential measurements of diester geminis at the concentration 6 × *cmc* was too low to get an intensity of the scattered light which would be high enough to determine particle electric mobility and subsequently calculate zeta potential. Therefore, zeta potential of the **e(s)** series was measured at the concentration 15 × *cmc*. As the results indicate (Figure 5B), there is no meaningful difference in zeta potential values for **a(s)** and **e(s)** series of gemini surfactants (the error bars to the zeta mean values of **a(s)** and **e(s)** surfactants almost overlap), especially in the region of medium spacer values where the mean zeta value lies between 15 and 25 mV. Certain increase in zeta potential is observed for gemini surfactants with the longest spacer value **s** = 8, especially for **e8** when its zeta potential value reaches almost +50 mV. This may be related to the above-mentioned aggregation tendency of **e8** molecules. 

### 2.5. 3D Models of Optimised Gemini Molecules

**Figure 7:**
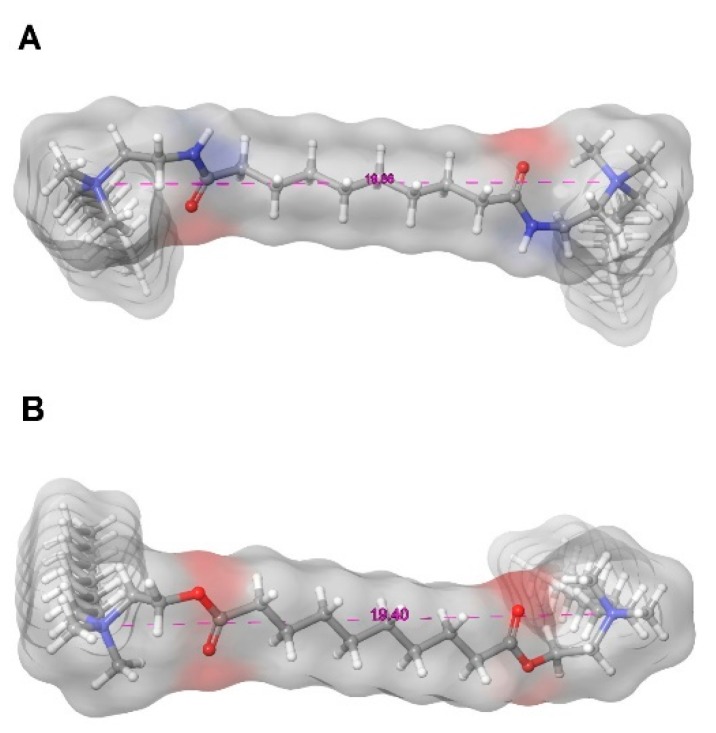
3D models of optimised **A**: **a8** and **B**: **e8** gemini molecules.

Optimised 3D conformers of **a8** and **e8** gemini molecules were constructed and are shown in Figure 7. Calculated N-to-N longitudinal size and van der Waals surface of **a8** and **e8** hydrophilic molecule parts are given in Table 4.

As results from Figure 7 and Table 4, the longitudinal size between ammonium nitrogen atoms of **e8** gemini molecule hydrophilic part containing two ester groups in the spacer is shorter than that of **a8** with two amide groups. This difference is also reflected in a smaller surface of the hydrophilic part of **e8** molecule. These findings correspond well with our previous 3D structural calculations and the determination of aggregation parameters of cationic surfactants with amide or ester group in the surfactant molecule structure. Higher flexibility of the ester group is responsible for the higher aggregation ability in the volume and a denser arrangement of surfactant molecules at the air-water interface of ester-based surfactants [33]. This may be the reason for the observed spacer flexibility of the sufficiently long gemini spacer of **e8** containing two ester groups, and consequently, for a stronger tendency to aggregate into larger structures, as documented particle size and aggregation number determination.

## 3. Materials and Methods 

### 3.1. Materials

Chemical formulas and related data of the series of diamide and diester gemini surfactants are shown in Section 2.1. The identity of the gemini surfactants was confirmed by elemental analysis (Flash 2000 organic elemental analyser, Thermo-Fischer Scientific (Waltham, MA, USA). Cetylpyridinium chloride (CPyCl) purchased from Sigma-Aldrich (Prague, Czech Republic) was used as a fluorescence quencher. Pyrene (Sigma-Aldrich, Prague, Czech Republic) was recrystallized prior to its use, and its ethanol stock solution (0.02 M) was kept in the dark. 

### 3.2. Time-Resolved Fluorescence Quenching

The fluorescence decay curves were recorded using the lifetime fluorescence spectrometer LifeSpec II (Edinburgh Instruments, Edinburgh, UK) at 340 nm excitation wavelength and 381 nm emission wavelength. The kinetic model of time-resolved fluorescence quenching considers that the non-exponentiality observed in the fluorescence decay is the result of the fact that each micelle does not contain an identical number of molecules of quencher. Instead, the quenchers are distributed over micelles according to a Poisson distribution. In the absence of quencher, the excited probe decays with a rate constant k = 1/τ_0_. The decay curve is single exponential for pyrene as the fluorescence probe. The presence of quencher affects the decay curve which is no longer single exponential and obeys the equation [34,35]:

(1) $$I\left(t\right)\;=\;I\left(0\right)exp\;\{-\frac{t}{\tau_{0}}\;-\;R\;\left[\;1-exp(-\;\frac{t}{\tau_{Q}}\;\right)]\}$$

*I*(t) and *I*(0) are the fluorescence intensities at time t and at the zero time, respectively. τ_0_ is the probe fluorescence lifetime in micellar environment, τ_Q_ is the probe fluorescence lifetime in the presence of the quencher. The meaning of the fitting parameter *R* is the ratio of quencher concentration *c*_Q_ to the concentration of micelles *c*_mic_, *R* = *c*_Q_/*c*_mic_. Concentration of surfactant molecules included in micelles in moles per liter is equal to the difference *c* − *cmc* where c is the molar surfactant concentration. Then, micelle aggregation number can be expressed as follows:

(2) $$N\;=\;\frac{c\;-\;cmc}{c_{mic}}\;=\;R\;\frac{c\;-\;cmc}{c_{Q}}\;$$

In the fluorescence quenching experiments, pyrene was used as a fluorescence agent and cetylpyridinium chloride as a quencher. To avoid the formation of pyrene excimers, pyrene concentration was kept at a low level, such that the ratio pyrene concentration/concentration of surfactant micelles in the solution was less than 0.05 [35]. The concentration ratio quencher concentration/concentration of surfactant micelles was close to 1, so that the quencher distribution is approximately one quencher molecule per micelle. The surfactant concentrations used were high enough to ensure that pyrene and the quencher were completely solubilized in the micelles. All measurements were performed at 25 °C.

### 3.3. Dynamic Light Scattering

The hydrodynamic diameter of **a(s)** and **e(s)** micelles was determined using a dynamic light scattering equipment (Brookhaven BI 9000 digital correlator, SM 200 goniometer (Brookhaven Instruments Corp., Holtsville, NY, USA)), argon laser (Lexel Laser, Fremont, CA, USA)). The argon laser at 514.5 nm wavelength was used as the incident light source. The intensity time fluctuations of the scattered light were detected at a scattering angle of 90° and a temperature 25 °C and autocorrelated in the BI 9000 correlator card where the time correlation function was built. The translation diffusion coefficient was calculated from the time correlation function using the method of cumulants. The method of cumulants was used for the calculation of the mean micelle diameter from the expansion of logarithm of time correlation function into a series up to the second quadratic term. The diffusion coefficient was determined from the correlation function decay rate and the hydrodynamic diameter *d* was calculated from the diffusion coefficient *D* using Stokes–Einstein formula:

*d* = k*T*/(3πη*D*) (3)

where η is solvent viscosity, k is the Boltzmann constant, and *T* is absolute temperature. Five independent measurements and calculations of time correlation function were carried out for each concentration and surfactant spacer length and the mean value and standard deviation of *d* was calculated from the set of five measurements. To determine particle size distributions, a numerical algorithm based on the inverse Laplace transformation was applied to the time correlation function. Micelle diameters were evaluated from the partition size distributions utilizing the CONTIN algorithm with the rejection probability parameter set to 0.5. Due to the larger sets of processed data, a custom application software written in Visual Basic was used for automated data format conversion from the measurement files.

### 3.4. Zeta Potential

Zeta potential measurements were performed with the Brookhaven BI ZetaPlus equipment which is based on the measurement of the electrophoretic mobility utilizing the Doppler frequency shift. Zeta potential values were calculated from the measured mobility using the Smoluchowski limit for the mobility vs. zeta potential relationship. The mean value was calculated from a statistical set of 20 zeta potential recordings at each surfactant concentration. The measurements were taken at 25 °C.

### 3.5. 3D Modelling of Gemini Molecules

The 3D structures of studied gemini surfactants were sketched utilising the Maestro interface of the Schrödinger software release 2017-1 Maestro software (see https://www.schrodinger.com/maestro for details, Schrödinger LLC, New York, NY, USA). Single point energy minimization using OPLS force field *in vacuo* was performed. The conformational analysis showed that the straight conformation of gemini spacer with the intramolecular electrostatic interaction between positive ammonium groups and a polar ester or amide group was found to be optimal.

## Figures and Tables

**Table 1:** *Cmc* values and molar weight *M*_r_ of **a(s)** and **e(s)** gemini surfactants.

Surfactant	*M*_r_	*cmc*	Surfactant	*M*_r_	*cmc*
		10^−3^M			10^−3^M
**a1**	742.80	1.50 ± 0.09	**e2**	758.79	1.52 ± 0.12
**a2**	756.82	1.26 ± 0.11	**e3**	772.82	0.57 ± 0.11
**a4**	784.88	1.36 ± 0.06	**e4**	786.85	0.86 ± 0.08
**a7**	826.96	1.01 ± 0.04	**e6**	814.90	0.53 ± 0.09
**a8**	840.98	0.74 ± 0.06	**e7**	828.93	0.47 ± 0.07
			**e8**	842.95	0.29 ± 0.10

**Table 2:** Values of fitting parameter *R*, micelle aggregation number *N* of diamide gemini surfactants **a(s)** at the concentrations 4 × *cmc* to 12 × *cmc*, as determined from time-resolved fluorescence quenching measurements.

*c/cmc*	a1		a2		a4		a7		a8	
	*R*	*N*	*R*	*N*	*R*	*N*	*R*	*N*	*R*	*N*
4	1.861 ± 0.020	35.8 ± 0.4	1.948 ± 0.019	31.5 ± 0.3	1.151 ± 0.013	20.1 ± 0.2	1.681 ± 0.035	21.7 ± 0.5	0.639 ± 0.011	14.3 ± 0.2
6	1.729 ± 0.018	37 ± 0.4	1.693 ± 0.017	30.4 ± 0.3	1.091 ± 0.012	21.1 ± 0.2	1.635 ± 0.037	23.5 ± 0.5	0.601 ± 0.015	14.9 ± 0.4
8	1.690 ± 0.021	38 ± 0.5	1.688 ± 0.020	31.9 ± 0.4	1.056 ±0.012	21.5 ±0.2	1.654 ± 0.040	25 ± 0.6	0.623 ± 0.011	16.2 ± 0.3
10	1.661 ± 0.018	38.4 ± 0.4	1.598 ± 0.020	31 ± 0.4	1.013 ± 0.012	21.2 ± 0.3	1.670 ± 0.041	25.9 ± 0.6	0.606 ± 0.016	16.2 ± 0.4
12	1.638 ± 0.019	38.5 ± 0.5	1.627 ± 0.018	32.2 ± 0.3	1.006 ± 0.012	21.4 ± 0.3	1.807 ± 0.051	28.6 ± 0.8	0.639 ± 0.019	17.4 ± 0.5

**Table 3:** **A**: Values of fitting parameter *R*, micelle aggregation number *N* of diester gemini surfactants **e(s), s** = 2 to 7, at the concentrations 4 × *cmc* to 12 × *cmc*, and surfactant **e8** at the concentrations 2 × *cmc* to 6 × *cmc* as determined from time-resolved fluorescence quenching measurements.

*c/cmc*	e2		e3		e4		e6		e7		*c/cmc*	e8	
	*R*	*N*	*R*	*N*	*R*	*N*	*R*	*N*	*R*	*N*		*R*	*N*
4	1.077 ± 0.015	24.6 ± 0.3	0.901 ± 0.013	23 ± 0.3	1.177 ± 0.015	22.8 ± 0.3	0.636 ± 0.012	15.2 ± 0.3	0.576 ± 0.016	12.2 ± 0.3	2	1.177 ± 0.074	10.2 ± 0.6
6	0.927 ± 0.023	23.5 ± 0.6	0.892 ± 0.016	25.2 ± 0.4	0.999 ± 0.020	21.6 ± 0.4	0.614 ± 0.022	16.3 ± 0.6	0.600 ± 0.017	14.1 ± 0.4	3	1.661 ± 0.040	19.2 ± 0.5
8	0.903 ± 0.018	24 ± 0.5	0.785 ± 0.022	23.3 ± 0.7	0.971 ± 0.022	22 ± 0.5	0.598 ± 0.020	16.6 ± 0.5	0.594 ± 0.022	14.6 ± 0.5	4	1.770 ± 0.069	23 ± 0.9
10	0.891 ± 0.020	24.4 ± 0.5	0.805 ± 0.027	24.6 ± 0.8	0.932 ± 0.018	21.7 ± 0.4	0.623 ± 0.029	17.8 ± 0.8	0.588 ± 0.014	14.9 ± 0.3	5	2.102 ± 0.065	29.2 ± 0.9
12	0.909 ± 0.021	25.3 ± 0.6	0.805 ± 0.027	25.1 ± 0.8	0.947 ± 0.023	22.5 ± 0.6	0.604 ± 0.020	17.6 ± 0.6	0.600 ± 0.021	15.5 ± 0.5	6	2.322 ± 0.079	33.6 ± 1.1

**Table 4:** Values of N-to-N longitudinal size and van der Waals surface of **a8** and **e8** hydrophilic molecule parts calculated by the OPLS force field using the Schrödinger Maestro software.

	N-to-N distance	Surface
	Å	Å^2^
**a8**	19.86	849.83
**e8**	19.40	839.65
